# Optimal exercise modalities and doses for therapeutic management of osteoarthritis of the knee

**DOI:** 10.3389/fragi.2025.1458983

**Published:** 2025-02-04

**Authors:** Max Whitfield, Owen W. Tomlinson

**Affiliations:** University of Exeter Medical School, Faculty of Health and Life Sciences, University of Exeter, Exeter, United Kingdom

**Keywords:** aerobic training, resistance training, running, high-intensity, low-intensity, dose-response, physical activity, Degeneration

## Abstract

Osteoarthritis (OA) is a progressive osteoarticular disease affecting the whole joint. In the United Kingdom, OA is the most prevalent joint disease, with knee osteoarthritis (KOA) being the most common type of OA. Key symptoms of KOA include knee pain, stiffness, and loss of physical function. Different types of exercise can be performed in people with KOA, which exert different magnitudes of impact forces on the knee joint, whereby the National Institute for Health and Care Excellence (NICE) OA guidelines in fact recommend exercise as the core form of therapeutic OA management. However, the optimal type or dose – the cumulative intensity, duration, and frequency – of therapeutic exercise that most effectively provides KOA management is not currently known. This review aims to summarise and compare the literature, discussing optimal exercise modalities and doses for the management of KOA. All exercise modalities proved similarly beneficial at managing KOA with comparable improvements to knee pain, stiffness, and physical function, therefore with no optimal exercise modality identified. Benefits to KOA management was observed in everyone, including the elderly, obese, and those with severe KOA. Although, in those with severe KOA, walking was observed to only prevent further deterioration, rather than induce any symptomatic improvement. Furthermore, there was minimal difference between exercise modalities in relation to the improvements not only in KOA symptoms, but also modifiable KOA risk factors, adherence, adverse events, and QoL. Assessment of the dose-response relationship of each exercise modality showed that any dose of regular exercise was effective. Although, moderate intensity exercise performed three times weekly for 20-to-60-min appears optimal for KOA symptom control following most exercise modalities. Therefore, those with KOA should be encouraged to continue whatever exercise they currently do as should effectively manage symptoms, regardless of the modality or dose. However, those who do no exercise should use the present review in collaboration with clinicians via shared decision making to create a holistic exercise prescription. In summary, this review contributes to the literature through comprehensive discussion of different exercise modalities and doses in managing not only KOA symptoms, but modifiable KOA risk factors, exercise adherence, adverse events, and QoL. Additionally, summarised findings are discussed to give practical exercise recommendations to promote effective KOA management and recommendations for ongoing research.

## 1 Introduction

Osteoarthritis (OA) is a progressive and degenerative osteoarticular disease negatively affecting the whole joint, including the cartilage, synovium, joint capsule, subchondral bone, and the intra- and peri-articular ligaments and muscles (Poole, 2012; Loeser et al., 2012). Osteoarthritis is the most common joint disease in the United Kingdom with an increasing prevalence, currently affecting 8.75 million people aged over 45 years old (Arthritis Research UK, 2013). The knee is the joint most affected by OA, with 18% of people over 45 years old suffering from knee osteoarthritis (KOA) (Arthritis Research UK, 2013).

### 1.1 Symptoms and diagnosis of knee osteoarthritis

Physical symptoms of KOA include knee pain, stiffness, and loss of physical function (Vignon et al., 2006; Kopec et al., 2007), with radiographic signs of KOA including loss of articular cartilage, joint space narrowing, osteophyte development, subchondral bone sclerosis, and synovitis (Stehling et al., 2010; Zhang et al., 2001). Within OA management, the Kellgren-Lawrence (KL) grading system is widely used to grade the radiographic severity of KOA using plain film radiography of the knee to assess for radiographic KOA signs to determine the individuals KL grade (Kohn et al., 2016). The resulting KL grade correlates with the severity of KOA, being ranked from 0–4; 0–1 indicates no/doubtful radiographic evidence of KOA, while 2, 3, and 4 indicates minimal, moderate, and severe KOA severity respectively (Kohn et al., 2016; Kellgren and Lawrence, 1957).

The significance of radiographic signs of KOA is disputed, as those with radiographic KOA often lack physical symptoms, creating uncertainty of KOA diagnosis in such individuals (Lawrence et al., 2004). Moreover, those with KOA symptoms similarly often lack radiographic KOA (Bedson and Croft, 2008), with KOA symptoms preceding the development of radiographic KOA by up to 3 years (Case et al., 2015). As a result, the National Institute for Health and Care Excellence (NICE) guidelines for the diagnosis and management of OA state that OA should be diagnosed clinically rather than explicitly relying upon radiographic imaging (National Institute for Health and Care Excellence, 2022). Those with KOA are at a greater risk of cardiovascular disease, diabetes, premature mortality (Vennu and Bindawas, 2014; Cereda et al., 2016), and a reduced health related quality of life (QoL) (Vennu and Bindawas, 2014; Øiestad et al., 2020). Consequently, OA is the 11th most debilitating disease worldwide (James et al., 2018), with a significant socioeconomic burden, reducing lifetime earnings of an individual with poorly managed OA by 33%–61% (Martins et al., 2021).

### 1.2 Progression and risk factors for knee osteoarthritis

**FIGURE 1:**
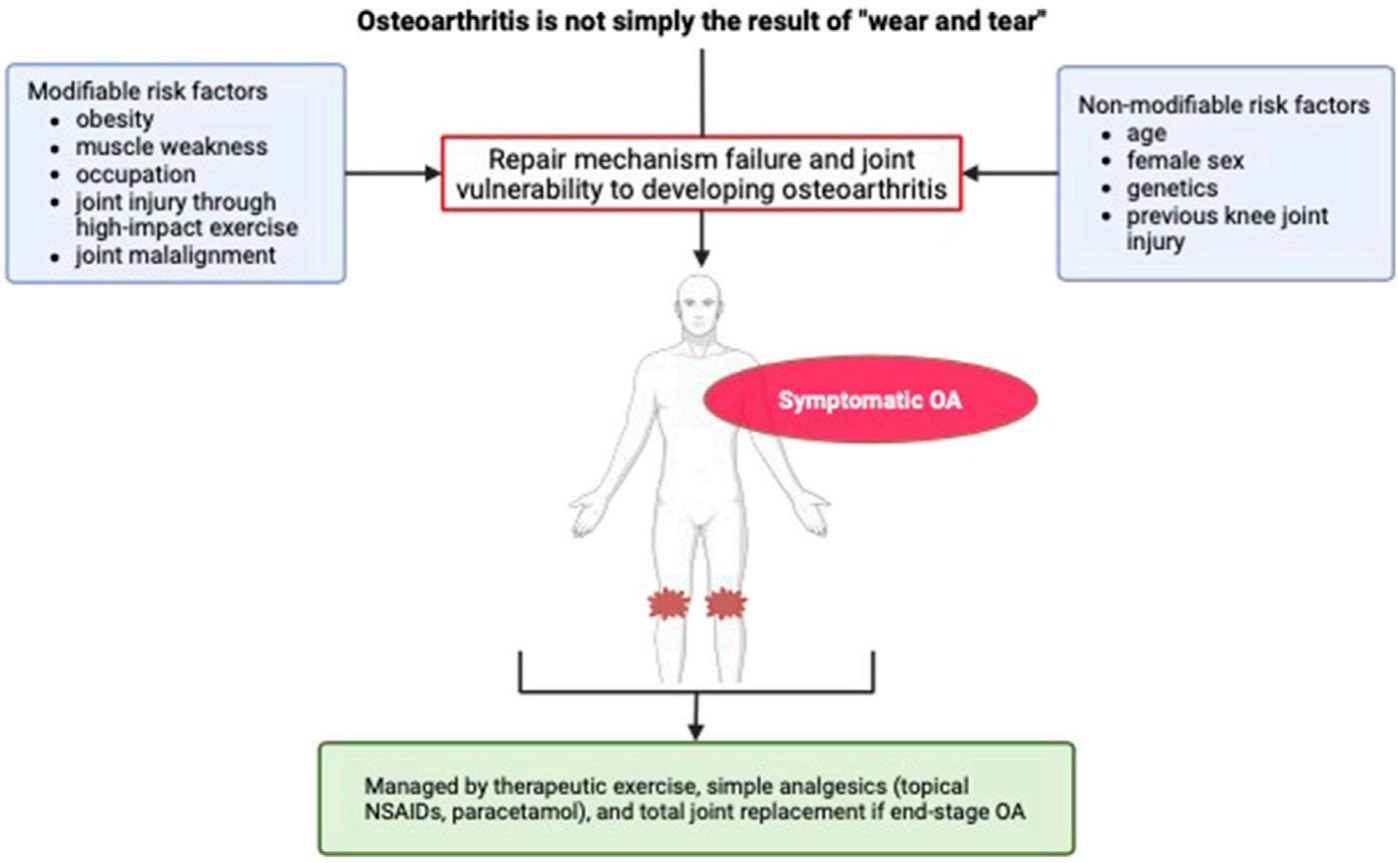
Risk factors and symptoms of knee osteoarthritis. OA, osteoarthritis; NSAID, non-steroidal anti-inflammatory drugs.

The development and progression of KOA has both modifiable and non-modifiable risk factors. The non-modifiable risk factors are increasing age, female sex (Glyn-Jones et al., 2015), genetics (Spector and MacGregor, 2004), and previous knee joint injury (Hunter and Eckstein, 2009). Whereas, modifiable risk factors include obesity (Garstang and Stitik, 2006), knee joint malalignment (Lim et al., 2008), quadriceps muscle weakness (Slemenda et al., 1997), physically demanding occupations (Yucesoy et al., 2015), and specific high impact sports that increase knee joint injury risk, such as football (Garstang and Stitik, 2006; Krajnc et al., 2010). Despite being poorly understood (Krajnc et al., 2010), KOA results from a complex multifactorial process (Glyn-Jones et al., 2015; Mobasheri and Batt, 2016) and not simply “wear and tear” (Thijssen et al., 2014) (Figure 1). The degenerative nature of KOA occurs due to impairment of cartilage, synovium, and subchondral bone repair mechanisms (Kisand et al., 2018; Li et al., 2013). Onset of this impairment likely results from greater vulnerability of the knee to developing OA due to the presence of the aforementioned risk factors (Glyn-Jones et al., 2015; Li et al., 2013). The strongest of the KOA risk factors implicated in its development is increasing age, followed by obesity and previous knee joint injury (Glyn-Jones et al., 2015).

### 1.3 Management of knee osteoarthritis

Currently, no effective treatment for KOA exists, with the aim of management being symptom control of knee pain, stiffness, and physical function (Chen et al., 2017). End-stage KOA often requires invasive surgical intervention, with the definitive surgical treatment being Total Knee Arthroplasty (TKA) (Chen et al., 2017; Price et al., 2018).

However, TKA is not without risks, with 15%–20% of patients postoperatively experiencing worsening of existing knee pain and physical function, with the additional risk of complications including infection (Price et al., 2018). Symptom control in those with KOA can be managed pharmacologically with simple analgesics including paracetamol and Non-Steroidal Anti-Inflammatory Drugs (NSAIDs) (National Institute for Health and Care Excellence, 2022). However, the NICE OA guidelines recommend therapeutic exercise as the primary management of all individuals with OA (National Institute for Health and Care Excellence, 2022). Regular exercise in those with KOA is seen to improve knee pain and physical function with a similar effect to simple analgesics, although without the risk of adverse pharmacological effects (Smith et al., 2013).

Adults are recommended to perform 150 min of moderate intensity exercise, or 75 min of more intense exercise, with two weekly exercise sessions aimed at improving muscle strength (UK Chief Medical Officers, 2019). Exercise is linked to lower rates of depression and anxiety (Kujala, 2010), and greater cardiovascular function, reducing the risk of cardiovascular disease and premature death (Nystoriak and Bhatnagar, 2018; Myers et al., 2004). In relation to KOA, exercise has potential to not only manage symptoms, but also reduce modifiable KOA risk factor burden, potentially slowing or preventing further disease progression, such as by lowering obesity rates (Wareham, 2007), while increasing quadriceps strength (Sutton et al., 2001), joint function and stability, thereby reducing injury risk (Fentem, 1994). Furthermore, exercise may reduce cartilage loss (Williams, 2013), with joint loading promoting cartilage nutrient supply, stimulating greater cartilage synthesis (Hall et al., 1991). In contract, physical inactivity and a lack of exercise is associated with accelerated cartilage degeneration possibly resulting from reduced joint loading (Esser and Bailey, 2011). Such potential of exercise at preventing KOA progression is evidenced by exercise being associated with a lower risk of requiring Total Knee Arthroplasty (TKA) due to severe KOA (Sharkey et al., 2000; Manninen et al., 2001), possibly reducing the healthcare burden of KOA. Therefore, regular exercise may preserve joint health and crucially reduce modifiable risk factors with the potential to prevent the development, progression, and healthcare burden of KOA.

Despite being recommended, only 4% of those with OA seen in a primary care setting perform regular exercise, compared to 31% and 26% who use paracetamol and topical NSAIDs respectively (Healey et al., 2018). Low uptake of therapeutic exercise may arise from uncertainty of the benefits of exercise for OA, with 18% of the public and healthcare professionals (HCPs) perceiving that running for exercise increases KOA progression (Esculier et al., 2018). While 3% of HCPs have recommended patients with KOA to stop running altogether (Esculier et al., 2018), whereas up to 50% of runners with OA reporting having been told by HCPs to reduce or limit how much they run (Hartwell et al., 2023). The negative perception of exercise may result from belief of the outdated “wear and tear” hypothesis of OA development and fear that exercise may exacerbate OA (Thijssen et al., 2014; Esculier et al., 2018). However, in contrast to such perceptions, running and other exercise types have been shown to lead to no greater prevalence of OA or rates of joint degeneration (Hartwell et al., 2023).

Different modalities of exercise exist, which exert different levels of compressive impact through load-bearing joints (Hunter and Eckstein, 2009). High-impact exercise modalities includes running, and high-load resistance exercise; while low-impact exercise modalities include walking, Tai Chi, cycling, swimming, aquatic exercise, and low-load resistance exercise (Hunter and Eckstein, 2009; Howe et al., 2011). Different modalities of therapeutic exercise in those with KOA are often perceived to theoretically have a differing impact on symptom control. High impact exercise may overload the vulnerable knee joint of someone with KOA, leading to symptom exacerbation (Hunter and Eckstein, 2009; Jan et al., 2009). While low-impact exercise exerts lower compressive forces at the knee joint, bringing the benefit of exercise, joint motion, and loading (Hunter and Eckstein, 2009; Jan et al., 2009), and possibly greater symptomatic relief (Vannini et al., 2016; Lo et al., 2022).

Within the literature, studies largely focus upon the clinical and functional outcomes of those with KOA such as knee pain, stiffness, and physical function, although fail to do so while comparing a wide range of different exercise types (Vannini et al., 2016; Bruyere, 2009). Furthermore, key patient centered outcomes indicative of an effective therapeutic intervention such as QoL, adherence, and modifiable KOA risk factor are often neglected (Sharkey et al., 2000). As a result, despite theoretical differences, the literature has failed to determine whether there is an optimal exercise type for KOA management (Sharkey et al., 2000; Vannini et al., 2016; Bruyere, 2009).

Exercise dose is measured by the intensity, duration, and frequency of exercise, and is a key variable of an effective therapeutic exercise prescription (Wasfy and Baggish, 2016). The relationship between dose and its outcome, whether therapeutic or harmful, is a dose-response relationship (Kilcoyne et al., 2013). This typically has two key thresholds, the minimum and the maximum effective dose, describing the smallest and largest dose where the therapeutic benefit outweighs any harmful effects. Between these thresholds is the optimal dose, which is the dose that most effectively improves beneficial outcomes with minimal harmful effects (Lee, 2007). In the literature, it is unknown whether an optimal exercise dose exists for the different exercise modalities in relation to knee pain, stiffness, or physical function in those with KOA (Sharkey et al., 2000; Alexander et al., 2021; Fransen et al., 2015).

This review aims to explore the influence of the modality and dose of exercise for the management of KOA to determine the optimal modality and dose of therapeutic exercise. The literature will be described to assess the influence of the different exercise modalities on knee pain, stiffness, physical function, and the additional patient-centered aspects of KOA management such as QoL, adherence, and influence on modifiable KOA risk factors. The dose-response relationship of each exercise modality will then be investigated to assess the optimal dose that most effectively manages knee pain, stiffness, and physical function of those with KOA. Finally, direct comparison of the relative effectiveness at improving the different aspects of KOA management will be performed to determine the optimal modality and dose of therapeutic exercise. Improving on the current understanding of the optimal modality and dose of therapeutic exercise for managing KOA; this review will better inform individuals on how best to manage their KOA safely and effectively.

## 2 Methodology

This paper presents a narrative literature review which without systematic selection criteria may introduce bias or inconsistency within the review. Literature relevant to this narrative review was identified using PubMed and Google Scholar search engines, whereby the terms “knee osteoarthritis” AND “exercise,” “exercise” AND “knee osteoarthritis” AND “dose,” “exercise” AND “knee osteoarthritis” AND “dose-response relationship” were searched. Literature was considered for inclusion as per Table 1 if it assessed at least one of the following outcomes in individuals with KOA: knee pain, knee stiffness, physical function, QoL, adherence, modifiable KOA risk factors, or adverse events. Literature that was not available in English or accessible via institutional open access arrangements was excluded.

**TABLE 1:** Population, intervention, comparison, outcome framework.

Population (1)	Intervention (7)	Comparison (3)	Outcome (7)
Knee Osteoarthritis	Walking	Usual care	Knee pain
	Tai Chi	Non-exercise control	Knee stiffness
	Running	Other exercise modalities	Physical function
	Cycling		Modifiable risk factors of knee osteoarthritis
	Swimming		Quality of life
	Aquatic exercise		Exercise adherence
	Resistance exercise		Adverse events

## 3 Exercise management of knee osteoarthritis

This narrative review consequently discusses in turn, various modalities of exercise for the management of KOA, acknowledging doses of each exercise and its therapeutic benefits. As previously mentioned, high-impact exercise exerts greater impact at the knee which may overload the vulnerable joint of those with KOA, possibly exacerbating symptoms (Hunter and Eckstein, 2009; Jan et al., 2009). While the low-impact exercise modalities, may be of greater benefit to KOA symptom management through lower impact joint loading and motion (Vannini et al., 2016; Lo et al., 2022). Theoretically low-impact exercise modalities may be of greater benefits, although it remains unclear whether there is an optimal exercise modality for KOA management (Sharkey et al., 2000; Vannini et al., 2016; Bruyere, 2009), prompting further research. Additionally, whether an optimal exercise dose exists for each modality will be determined, adding to the poor understanding of exercise dose in the management of KOA.

### 3.1 Walking

Walking is the most popular exercise modality in England, performed by 22.9 million adults two or more times per month (Sport England, 2023). Walking promotes mental wellbeing while reducing the risk of cardiovascular disease, cancer, obesity, and type 2 diabetes (National Institute for Health and Care Excellence, 2012), ultimately reducing all-cause mortality by 11% (Kelly et al., 2014). With a range of benefits and ease of accessibility, walking is recommended by NICE as an exercise modality suitable for everyone (National Institute for Health and Care Excellence, 2012). Walking will subsequently be discussed, determining its potential benefit and optimal therapeutic doses in managing KOA symptoms.

#### 3.1.1 Walking for symptom management

##### 3.1.1.1 Walking and pain

Knee pain in those with KOA has been shown to be reduced following walking. Using the “Western Ontario and McMaster Universities Osteoarthritis Index” (WOMAC) pain scale, walking reduced knee pain by 51%–77% over 6-to-12-week (Evcik and Sonel, 2002; Samut et al., 2015; Shahine et al., 2021), while on the Visual Analogue Scale (VAS) pain severity reduced by 35%–47% over a similar 6-to-12-week period (Evcik and Sonel, 2002; Samut et al., 2015; Bautch et al., 1997). Knee pain on the Arthritis Impact Measurement Scale (AIMS) reduced by 39% following 8-weeks of walking (Kovar et al., 1992). However, knee pain did not improve in those with a severe KL grade of KOA, but rather walking prevented further worsening of existing knee pain over 12 weeks (Wallis et al., 2017). Over an 18-month period, walking significantly reduced knee pain severity in elderly obese individuals with KOA (Messier et al., 1997).

Different pain types can be improved by walking, with the Intermittent and Constant Osteoarthritis Pain (ICOAP) questionnaire showing a ∼25% reduction in constant and intermittent knee pain (Beckwée et al., 2016). Moreover, in self-selected walkers with KOA over an 8-year period, a 40% reduction in self-reported frequency of new knee pain is experienced, although no improvements to existing pain are reported (Lo et al., 2022). However, as participants self-reported their pain and walking status, the direction of causation cannot be determined. This is because those with greater pain following walking are likely to stop walking thus being excluded from the walking group analysis, leaving only those who experience reduced knee pain in the walking group.

##### 3.1.1.2 Walking and stiffness

With relation to knee stiffness, this may also be reduced following walking in those with KOA. Walking over 6-to-12-week improved WOMAC stiffness scores by 47%–62% (Samut et al., 2015; Shahine et al., 2021), with significant improvement observed for up to 18-month of walking (Brosseau et al., 2012). However, despite an initial high adherence to walking at 3 months of 85%, adherence declined to 50% at 18 months (Ettinger et al., 1997). Such a fall in adherence would likely limit the long-term effectiveness of walking, with the most adherent walkers experiencing the greatest improvement in knee pain and physical function (Ettinger et al., 1997). However, it may be that walking was less effective at managing KOA symptoms in the individuals who were less adherent to walking, or that walking became less effective over time, therefore, driving the fall in adherence over time. Despite a single walking bout reducing knee stiffness (Wallis et al., 2015), over a 12-week period of walking in those with a severe KL KOA grade, knee stiffness did not improve (Wallis et al., 2017).

##### 3.1.1.3 Walking and physical function

Physical function in those with KOA can also be improved by walking, with AIMS physical function improving by 39% over 8-weeks (Kovar et al., 1992), with significant improvement on the Activities of Daily Living (ADL) scale also reported (Lin et al., 2022). While WOMAC physical function improved by 45%–63% over 12-weeks (Evcik and Sonel, 2002; Samut et al., 2015), with significant improvement remaining for up to 18-month of walking (Brosseau et al., 2012; Ettinger et al., 1997). However, despite greater physical function following a single walking bout (Wallis et al., 2015), 12-weeks of walking may not improve–but instead, maintain–physical function in those with a severe KL grade of KOA (Wallis et al., 2017).

In addition to global functioning scores, functional improvements are also seen via exercise tests. For example, distance covered during a 6-Minute Walk Test (6MWT) can be significantly increased by 18% (381 m vs. 451 m) following 8-weeks of walking, relative to a control group whose 6MWT distance fell by 5% (356 m vs. 339 m) in the same period (Kovar et al., 1992). Furthermore, walking speed increased by 8% following 18-month of walking (109.4 cm^.^s^−1^ vs. 117.7 cm^.^s^−1^) (Messier et al., 1997). Finally, functional muscle strength improved following 6-weeks of walking with significantly greater Sit-To-Stand-Test (STST) repetitions performed in 30-s (Samut et al., 2015).

##### 3.1.1.4 Walking and lifestyle

Additionally, walking showed the potential to reduce obesity of those with KOA, with 26% of participants reducing body weight following 12-weeks of walking, whereas no loss of body weight was seen in control groups (Evcik and Sonel, 2002). However, in two separate studies, one participant experienced falls, with one suffering a radial (Ettinger et al., 1997), and the other a hip fracture (Kovar et al., 1992), possibly indicating a greater fall risk with walking in those with KOA. However, falls are also reported in non-exercise control groups (Kovar et al., 1992), suggesting no greater fall risk in those performing therapeutic walking. Balance was also significantly improved by walking, possibly reducing fall risk of those with KOA (Messier et al., 2000). Finally, QoL was improved in walkers with KOA (Evcik and Sonel, 2002; Brosseau et al., 2012), with a 40% reduction in Centre for Epidemiologic Studies Depression (CES-D) score (Penninx et al., 2002).

Therefore, knee pain, stiffness, and physical function are improved by walking in those with KOA, including in those with obesity and older age (Samut et al., 2015; Shahine et al., 2021). However, in those with severe KOA, the only benefit of walking is the prevention of further deterioration of knee pain and physical function (Wallis et al., 2017).

#### 3.1.2 Walking: Dose-response relationship

Moderate intensity walking, performed for three 40-min sessions per week, over 18 weeks, improved both constant and intermittent ICOAP pain score by 24% (Beckwée et al., 2016). Walking for the same duration, frequency, and intensity, but over a longer 18-month period had no further effect on pain, despite initially improving pain intensity by 12% (Messier et al., 1997). However, pain frequency during walking reduced by 19%, with a 21% reduced intensity and 16% reduced frequency of knee pain during transfer activities at 3-months, with improvements maintained at 18-month (Messier et al., 1997).

A greater dose of three 60-min sessions per week of moderate intensity walking for 12 weeks reduced current VAS pain by 37% and VAS pain over the previous week by 35% (Bautch et al., 1997). Therefore, a greater dose may lead to greater pain reduction, although the use of different scoring systems may influence the results, preventing comprehensive conclusions being made. Low-intensity walking at 1.5–2.5 km.h^−1^ was compared to moderate intensity walking at 4.0–4.5 km.h^−1^, with both being performed for five 30-min sessions per week in those with KOA. It was seen that moderate-intensity walking resulted in a greater improvement in physical function than low-intensity walking (Lin et al., 2022). Further work from Rejeski et al. (1997), assessed the dose-response relationship between adherence, time spent walking and the outcomes of knee pain and physical function, whereby an optimal dose of three 35-min bouts of moderate intensity walking was found to most effectively manage knee pain and physical function in those with any severity of KOA (Rejeski et al., 1997).

A maximum tolerable dose of 70-min of moderate intensity walking per week over any frequency and duration of sessions has been identified in those with a severe KL grade of KOA, above which knee pain, stiffness, and physical function are exacerbated (Wallis et al., 2015). At this weekly dose of walking over 12-week, the worsening of knee pain, stiffness, and physical function is prevented in those with a severe KL grade of KOA (Wallis et al., 2017). Furthermore, non-linear modelling identified a U-shaped dose-response relationship between walking dose and knee pain, stiffness, and physical function (Wallis et al., 2015). This identified an optimal dose of 50-min per week of moderate intensity walking to most effectively managing knee pain, stiffness, and physical function in those with a severe KL grade of KOA (Wallis et al., 2015). Therefore, moderate intensity walking may be more beneficial than low intensity walking with an optimal moderate intensity dose of three 35-min bouts of walking per week for less severe KOA, but an optimal dose of 50-min per week in total for severe KOA. However, comparison to high-intensity walking is yet to be investigated in those with KOA.

### 3.2 Tai Chi

Tai Chi is a low-impact exercise modality where participants use controlled diaphragmatic breathing while performing different sequenced body movements (Wang et al., 2004; Kutner et al., 1997). Tai Chi is regarded as a mind-body exercise due to its proposed influence on promoting the mind-body interaction (Kutner et al., 1997). As such, balance, coordination, and strength are improved following regular Tai Chi, with a reduced risk of falls and rates of depression (Kutner et al., 1997; Kolasinski et al., 2019). In addition, Tai Chi is a strongly recommended exercise modality in the guidelines for knee and hip OA produced by the American College of Rheumatology and the Arthritis Foundation (Kolasinski et al., 2019). Therefore, Tai Chi in the management of KOA will be discussed to assess its therapeutic benefit and optimal dose.

#### 3.2.1 Tai Chi for symptom management

##### 3.2.1.1 Tai Chi and pain

Knee pain in those with KOA has been seen to improve following periods of Tai Chi. Pain measured via the WOMAC scale improved by 66%–75% over 12 weeks (Wang C. et al., 2016; Wang et al., 2009), 39%–76% over 24 weeks (Wang C. et al., 2016; Wang et al., 2009; Zhu et al., 2016; Hu et al., 2019), and 54%–55% over 52 weeks (Wang C. et al., 2016; Wang et al., 2009). Similarly, VAS knee pain reduced by 48%–71% over 12 weeks (Wang et al., 2009; Brismée et al., 2007), 40%–56% over 24 weeks (Wang et al., 2009; Hu et al., 2019), and 39% over 52 weeks (Wang et al., 2009). When measured on the Knee injury and Osteoarthritis Outcome Score (KOOS), knee pain in those with KOA reduced by 15% over 12 weeks of Tai Chi, although 7% of participants experienced knee pain exacerbation. However, 25% of the non-exercising control experienced knee pain exacerbation, suggesting a reduced incidence of pain exacerbation in those with KOA following Tai Chi (Liu et al., 2019).

##### 3.2.1.2 Tai Chi and quality of life

When examined via the KOOS, QoL has been shown to improve by 25% (Liu et al., 2019), with a 47%–54% reduction on the CES-D scale (Wang et al., 2009), and a 14%–28% reduction on the Beck Depression Inventory-II (BDI-II) score (Wang C. et al., 2016), indicative of improved QoL. Greater social interaction, QoL, and wellbeing following Tai Chi is believed to reduce fear of knee pain in those with KOA, leading to better pain control (Yau, 2008). However, such benefit may arise from Tai Chi often being performed in a group setting rather than at home, and thus exact causation is not wholly clear and warrants further investigation. Such benefits of Tai Chi over 6-weeks of group sessions were maintained over a following 6-weeks of home Tai Chi training, although any further benefit is attenuated (Brismée et al., 2007). Furthermore, the observed additional benefit of group training may not be exclusive to Tai Chi, but also other forms of group exercise, demonstrating an additional area that requires further investigation, as highlighted in the NICE OA guidelines (National Institute for Health and Care Excellence, 2022).

##### 3.2.1.3 Tai Chi and stiffness

Tai Chi reduces knee stiffness in those with KOA, improving WOMAC stiffness score by 69% at 12 weeks (Brismée et al., 2007), and 51%–61% at 24 weeks (Wang et al., 2009; Zhu et al., 2016; Hu et al., 2019), with knee stiffness remaining improved for up to 48 weeks of Tai Chi (Brismée et al., 2007). Within these studies, adherence to Tai Chi ranged from 74%–90% over 12-to-24-weeks (Wang et al., 2009; Zhu et al., 2016; Hu et al., 2019; Brismée et al., 2007).

##### 3.2.1.4 Tai Chi and physical function

Tai Chi also leads to greater physical function in those with KOA, improving WOMAC physical function score by 67%–72% at 12 weeks (Wang C. et al., 2016; Wang et al., 2009), 36%–93% at 24 weeks (Wang C. et al., 2016; Wang et al., 2009; Zhu et al., 2016; Hu et al., 2019), and 57%–58% at 52 weeks (Wang C. et al., 2016; Wang et al., 2009).

Functional measures of physical function also improved, with a 7%–11% increase in 6MWT distance over 12-to-24-weeks, increasing by 29–46 m (Wang C. et al., 2016; Wang et al., 2009). In addition, balance in elderly individuals with KOA improved by 9% (Wang et al., 2009; Ribeiro and Oliveira, 2007), likely reducing the fall risk of those with KOA. Greater functional muscle strength is indicated by increased STST and 30-s arm curl repetitions (Chen et al., 2021). However, Tai Chi had no effect on reducing obesity, lacking the ability to modify a key KOA risk factor (Wang et al., 2009).

The wide range of improvement noted following 24-weeks of Tai Chi, particularly for knee pain and physical function, may be due to differences in participant KOA severity. The participants in one study included 30% who were “doubtful” as to having KOA, 52% having “mild,” 17% “moderate,” but none with severe KL grades of KOA (Wang et al., 2009). However, two of the other studies included 0%–7% doubtful, 20%–36% mild, 35%–40% moderate, and 16%–45% with a severe KL grade of KOA (Wang C. et al., 2016; Wang et al., 2009). Therefore, the difference in KOA severity of participants between the studies likely influenced participants baseline knee pain and physical function, and such the subsequent degree of improvement. Despite this, Tai Chi has been shown to improve knee pain, stiffness, and physical function in those with KOA, including those with obesity, older age, and severe KOA (Wang C. et al., 2016; Wang et al., 2009; Hu et al., 2019; Chen et al., 2021), demonstrating its benefit to managing KOA.

#### 3.2.2 Tai Chi: dose-response relationship

Within the literature, the definitive exercise intensities of Tai Chi remain to be defined. Tai Chi can be performed with different training styles and postures which can in turn, vary its exercise intensity, although, these remain to be extensively researched to provide its definitive exercise intensity (Huang et al., 2021). However, following the investigation of the cardiorespiratory and metabolic energy requirement response, one study showed Tai Chi to be a low-intensity form of exercise (Smith et al., 2015). Therefore, to assess the dose-response relationship of Tai Chi in the present review, the exercise intensity of Tai Chi is assumed to be constantly undertaken at a low-intensity, with its dose therefore varying by session duration and frequency.

Tai Chi for two weekly 60-min sessions improved VAS pain by 40%, and WOMAC pain, stiffness, and physical function scores by 76%, 58%, and 93% respectively (Hu et al., 2019). While Tai Chi for three weekly 60-min sessions improved WOMAC pain by 39%, stiffness by 50%, and physical function by 36% over 24-weeks (Zhu et al., 2016). Therefore, the lower dose of two weekly 60-min sessions may be more beneficial for those with KOA. However, two weekly 60-min Tai Chi sessions combined with 20-min of daily home practice of Tai Chi for 12-weeks improved WOMAC pain, stiffness, and physical function by 66%–71%, 69%, and 67%–72% respectively (Wang C. et al., 2016; Wang et al., 2009), and VAS pain by 71% (Wang C. et al., 2016). These conflicting results suggest that a lower weekly dose may lead to a marginally greater WOMAC score improvement, but smaller improvement in VAS pain score. The significance of such variation between studies and doses is difficult to establish due to the likely variation of participant characteristics and possibly the intensity of the Tai Chi exercise itself. Furthermore, differences in the components of the WOMAC and VAS pain scores may further complicate the significance of the findings.

Moreover, Lee et al. (2018) demonstrated a linear dose-response relationship indicating that greater adherence to two weekly 60-min sessions combined with 20-min of daily home Tai Chi leads to greater improvement of knee pain and physical function over 11-weeks. Therefore, suggesting that higher doses of Tai Chi may be more effective at providing KOA symptom control.

However, from 8-weeks onwards the linear dose-response relationship began slightly to level off, indicating that over longer period Tai Chi may become less effective (Lee et al., 2018). Although, this loss of effectiveness may result from those with KOA becoming accustomed to the stimulation given by the same dose and training style or posture of Tai Chi over time. Rather, to optimise the benefits of Tai Chi over longer periods of time it may be necessary to progressively increase its dose or change training style or posture.

Consequently, the literature is conflicted regarding whether higher Tai Chi doses are more effective, with the optimal dose remaining to be found. Therefore, Tai Chi requires further investigation, particularly over longer time periods where progressively greater doses or differing training styles or postures may be more effective.

### 3.3 Running

Running is a popular exercise modality in England, performed by 5.9 million adults two or more times per month (Sport England, 2023). Runners experience a 25%–40% lower risk of premature death, with on average a 3-year greater life expectancy than non-runners, likely due to significantly reduced risks of cardiovascular disease and some cancers in runner (Feldman, 2018; Lee et al., 2017). Furthermore, being easily accessible and convenient, running therefore mitigates some potential barriers to performing exercise (Lee et al., 2017).

However, running is high impact and has long been perceived to increase OA prevalence and progression (Esculier et al., 2018). Although, recent studies have shown that running does not increase OA prevalence or rates of joint degeneration (Hartwell et al., 2023). Therefore, being a popular, easily accessible exercise modality with significant health benefits, running for the management of KOA will be discussed, assessing its therapeutic benefit and possible optimal dose.

#### 3.3.1 Running for symptom management

##### 3.3.1.1 Running and pain

In the literature, the influence of running on symptom control in those with KOA remains to be extensively researched, having only been investigated in two studies. Over a 2-year period, self-selected runners with KOA reported a 2.2% reduction in the frequency of new knee pain, and a 10.9% reduction in the frequency of existing knee pain compared to non-runners with KOA (Lo et al., 2018). However, those with KOA who experience worse symptom control following running may stop running and subsequently be excluded from study analysis; potentially removing those with KOA who experience greater knee pain with running.

Therefore, the use of self-selected runners to assess self-reported pain prevents the direction of causation from being determined. However, this influence of symptoms on self-selected runners may be protective, with symptom exacerbation possibly leading to self-selection of a lower running dose that improves knee pain in those with KOA (Lo et al., 2018). Although, this fails to determine whether non-runners with KOA who begin running would see the same benefit.

##### 3.3.1.2 Running and physical function

Physical function on the ADL scale significantly improved by 41% following a 6-month period of running in those with a mild to moderate KL severity grade of KOA, with no adverse events reported (Lin et al., 2022). However, running may not bring the same benefit to those with severe KOA, as these individuals were excluded from the study. Participants were also randomised to walking, which at moderate intensity was more effective than running at improving physical function (Lin et al., 2022).

However, the study failed to investigate knee pain or stiffness preventing further conclusions of the influence of running on those with KOA. Furthermore, walking has been demonstrated to be beneficial in those with severe KOA (Wallis et al., 2017), while running remains to be investigated in those with severe KOA. Additionally, comparison of self-selected runners and non-runners with KOA showed no difference in BMI, indicating that running may not reduce obesity rates in those with KOA (Lo et al., 2018).

Running may improve knee pain and physical function, although with limited investigation, definitive conclusions regarding its influence on KOA management cannot be made. Therefore, future prospective cohort studies are recommended to investigate how running may manage KOA symptoms, alongside QoL, BMI, adherence, and muscle strength in those with KOA.

#### 3.3.2 Running: dose-response relationship

A dose of five weekly 30-min running sessions over 6 months improved physical function of those with KOA (Lin et al., 2022). However, the exercise intensity was poorly measured, with participants running at 6.0–7.5 km/h (Lin et al., 2022), which is not a subject-specific measure of exercise intensity, and therefore precise running dose cannot be assessed.

Cartilage volume transiently decreases during running as joint loading expels water from the cartilage (Crowder et al., 2021) and the return to baseline volume following running has been observed to be impaired in those with KOA (Esculier et al., 2019). Therefore, runners with KOA may require a longer recovery to allow for cartilage recovery (Esculier et al., 2019), particularly at higher running doses to prevent potential joint overloading and symptom exacerbation.

However, following a relatively high running dose, physical function did improve (Lin et al., 2022), although the same may not be true for knee pain and stiffness, warranting further investigation. Lo et al. (2018) found that a self-selected running dose improved physical function, however, specific data of participants running dose was not collected, preventing assessment of an optimal dose. Although, a self-selected dose could be protective, with runners likely selecting a lower running dose if symptoms become exacerbated (Lo et al., 2018).

Therefore, with minimal research in the literature, the optimal dose of running is yet to be found. However, a self-selected dose or a dose of five 30-min running sessions per week may possibly reduce knee pain and improve physical function, and therefore be a safe running doses for KOA management. Although, runners with KOA must be aware of the potential that they may require a longer recovery period between each run, particularly at higher doses.

### 3.4 Cycling

Cycling is a popular exercise modality, with 6.4 million adults cycling two or more times per month in England (Sport England, 2023). Regular cycling can improve cardiorespiratory fitness and reduce the incidence of obesity (Oja et al., 2011), with an hour of moderate intensity cycling per day leading to a 20% risk reduction in both all-cause and cancer mortality in women (Matthews et al., 2007). As such, cycling is a recommended exercise modality by NICE as being suitable for everyone (National Institute for Health and Care Excellence, 2012).

Furthermore, cycling may reduce knee joint inflammation and pain in those with KOA, as cycling involves repeated low impact joint movement believed to improve cartilage blood flow and nutrient delivery (Luan et al., 2020). Therefore, cycling as an exercise modality to manage KOA will be discussed to determine its therapeutic benefit and optimal dose.

#### 3.4.1 Cycling for symptom management

##### 3.4.1.1 Cycling and pain

Cycling may improve knee pain of those with KOA. Over 10-weeks of cycling, AIMS knee pain score improved by 12%–29% (Mangione et al., 1999). While over 12-weeks of cycling, knee pain reduced by 10%–19% on the KOOS pain scale (Liu et al., 2019; Salacinski et al., 2012); 15% on the WOMAC pain scale; and by 50% both at rest, and during walking on the VAS scale (Salacinski et al., 2012). Therefore, knee pain is improved following a 10-to-12-week period of cycling, although the magnitude of such improvement differs, with this variance likely due to the use of different scoring systems.

However, despite some improvements, 7%–30% may experience greater pain with cycling (Liu et al., 2019; Salacinski et al., 2012; Silvis et al., 2016; Keogh et al., 2018; Alkatan et al., 2016). One study reported that whilst 28% of those with KOA report worsening knee pain following cycling, 70% of people reported improved knee pain (Salacinski et al., 2012). Furthermore, a greater number of non-exercise control group participants have reported worsening knee pain than those performing cycling, at 22% and 7% respectively (Liu et al., 2019). The literature shows that the majority of those with KOA experience improved knee pain, while non-exercisers with KOA experience higher rates of worsening knee pain.

##### 3.4.1.2 Cycling and physical function

Cycling also improves knee stiffness, although has only been investigated in two studies. Following 12-weeks of cycling, WOMAC stiffness score decreased by 19%–30%, indicating improved knee stiffness (Salacinski et al., 2012; Alkatan et al., 2016). While over 12-weeks physical function improved by 15%–16% on the KOOS scale (Zhu et al., 2016; Crowder et al., 2021), and 16%–26% on the WOMAC scale (Salacinski et al., 2012; Alkatan et al., 2016).

Therefore, cycling improved physical function in those with KOA, with better physical function also indicated by a 7%–11% increase in 6MWT distance (Mangione et al., 1999; Alkatan et al., 2016), and a 4%–13% increase in walking speed (Mangione et al., 1999; Salacinski et al., 2012; Alkatan et al., 2016). Muscle strength of those with KOA improved following cycling with a 13%–18% increase in 30-s STST repetitions (Keogh et al., 2018), and a 13%–20% and 16%–22% increase in knee extensor, and flexor strength respectively (Alkatan et al., 2016).

When considering body composition, cycling significantly reduced body mass (84.5 kg–83 kg); visceral adipose tissue mass (3.3 kg–3.2 kg), and both hip and waist circumference of those with KOA over 12 weeks, alongside reducing (non-significantly) BMI by 1.9% (Alkatan et al., 2016). Therefore, whilst cycling failed to reduce obesity over 12-weeks, it may have the potential to do so over longer periods of time in those with KOA.

Adherence to cycling was 88%–97% over 8-to-12 weeks (Mangione et al., 1999; Keogh et al., 2018; Alkatan et al., 2016). This high adherence level may result two of the studies performing cycling in a supervised-group setting which may improve adherence (Mangione et al., 1999; Alkatan et al., 2016). However, Keogh et al. (Keogh et al., 2018) demonstrated high adherence to unsupervised home-based cycling, although adherence was self-reported in this study, thus possibly introducing a self-report bias into the results.

##### 3.4.1.3 Cycling and quality of life

Quality of life has also been improved by cycling, with KOOS QoL score increasing by 12%–22% (Liu et al., 2019; Salacinski et al., 2012), and improving the mental and physical domains of the 36-item Short Form (SF-36) questionnaire by 22%, and 35% respectively (Alkatan et al., 2016). Therefore, cycling improves knee pain, stiffness, and physical function in those with KOA, including in the elderly, obese, and those with severe KOA (Mangione et al., 1999; Silvis et al., 2016; Alkatan et al., 2016), with improved QoL, greater muscle strength, and potentially reduced obesity over longer time periods.

#### 3.4.2 Cycling: dose-response relationship

Moderate intensity cycling performed twice weekly for 60-min per session, over 12-weeks, improved KOOS score by 19%, and WOMAC pain, stiffness, and physical function scores by 15%, 19%, and 16% respectively (Salacinski et al., 2012). Moreover, when moderate intensity cycling is performed for three 40-min sessions per week over 12-weeks, WOMAC pain, stiffness, and physical function scores improved by 42%, 30%, and 26% respectively, with total WOMAC score improving by 30% (Alkatan et al., 2016). While four weekly cycling sessions improved total WOMAC score by 34% (Keogh et al., 2018), indicating that a higher session frequency, but shorter session duration, may be more effective. Whereas a greater dose of five-weekly, 60-min moderate-intensity sessions for 12-weeks improved KOOS pain score by 10% (Liu et al., 2019), indicating no greater benefit of a higher dose.

High intensity cycling in the form of High Intensity Interval Training (HIIT) performed for 10 min, four times per week, improved total WOMAC score by 41% (Keogh et al., 2018). However, three-weekly sessions of 25-min of either low- or high-intensity cycling over 10-week improved AIMS pain score by 29% and 12% respectively (Mangione et al., 1999), indicative of more effective pain control following low intensity cycling. Therefore, differing results are seen regarding high intensity cycling with reports of greater total symptom control but possibly less effective improvement to pain control. As a result, the optimal cycling dose may possibly be four weekly 20-min sessions of moderate intensity cycling; however higher intensities may be better and warrant further investigation.

### 3.5 Swimming

Swimming is performed two or more times per month by 3.8 million adults in England (Sport England, 2023). Similar to other exercise modalities, swimming benefits health with improved cardiorespiratory fitness (Chase et al., 2008) while being a low impact non-weight bearing modality (Lo et al., 2019). Swimming is associated with a significant 28% reduction in all-cause mortality risk, and a 41% reduction in cardiovascular mortality (Oja et al., 2011).

Therefore, swimming is often widely recommended for those with KOA (Lo et al., 2019), particularly those at greater risk of overloading the knee when weight bearing (Alkatan et al., 2016), such as those with obesity. Therefore, swimming will subsequently be discussed, determining its therapeutic benefit on symptomatic KOA and optimal dose.

#### 3.5.1 Swimming for symptom management

##### 3.5.1.1 Swimming and pain

Swimming in those with KOA has been observed to improve knee pain, whereby self-selected swimmers with KOA have significantly lower knee pain frequency and symptomatic KOA prevalence (Lo et al., 2019). However, this level of significance is lost when accounting for KOA risk factors such as increased age, female sex, obesity, previous knee injury, and individuals’ leisure physical activity levels (Lo et al., 2019). Therefore, swimmers may be generally physically active alongside swimming-specific activities which may drive the benefits, rather than the benefits arising from swimming alone. However, the study did not publish the data regarding the background physical activity levels of the participants and therefore this pathway cannot be fully concluded. The retrospective study design and use of self-selected swimmers also prevents the direction of causality from being identified (Lo et al., 2019).

##### 3.5.1.2 Swimming and physical function

Swimming over 12-weeks in obese individuals with a mild to moderate KL grade of KOA severity has been shown to improve WOMAC pain, stiffness, and physical function by 39%, 32%, and 44% respectively, with 6MWT distance also increasing by 6% (556 m–589 m), indicating improved physical function (Alkatan et al., 2016). However, a non-exercise control group was not included so the improvements may not entirely result from swimming; rather, the improvements could arise from the participants performing group swimming whereby the social interaction may drive the benefits–similar to group Tai Chi training as previously discussed. Although, this study did include a cycling group which provided similar symptom control to swimming (Alkatan et al., 2016).

In addition, swimming has significantly reduced total body mass from 92 kg to 89.4 kg; visceral adipose tissue mass from 3.4 kg to 3 kg; and hip and waist circumference although BMI failed to reduce significantly, only falling by 2% over the 12-week period (Alkatan et al., 2016). Therefore, swimming may have the potential to reduce obesity over longer time periods, but not over a 12-week timeframe, similar to cycling. Quality of life of those with KOA was also improved following swimming, with a 22% and 38% improvement in the mental and physical components of SF-36 respectively (Alkatan et al., 2016). Muscle strength also improved, with a 16%–24% and 19%–29% increase in knee extensor and flexor strength respectively (Alkatan et al., 2016). Although, the technique requirement of swimming may be a barrier to some, with one study reporting a participant with no swimming experience who only with swim teaching was able to participate (Alkatan et al., 2016). However, this may not be an economically viable option, although, the health economics of such intervention remains to be investigated.

Therefore, swimming may improve knee pain, stiffness, and physical function with similar benefit to cycling, however, with limited investigation within the literature, remains to be investigated in those with severe KOA. Furthermore, poor study design of the current literature, including the retrospective study designs (Lo et al., 2019), and lack of control groups (Alkatan et al., 2016), prevents swimming from conclusively being an effective type of therapeutic exercise for KOA management.

#### 3.5.2 Swimming: dose-response relationship

Three-weekly 45-min swimming sessions at a moderate intensity over 12-week improved WOMAC knee pain by 39%, stiffness by 32%, and physical function by 44% (Alkatan et al., 2016). Additionally, at this dose the adherence was high at 98%, possibly due to the swimming being performed in a group setting, and at no economic cost to the participants (Alkatan et al., 2016). Furthermore, a larger number of years an individual has swam for resulted in a significantly greater reduction in knee pain frequency and symptomatic KOA prevalence (Lo et al., 2019). However, the significance of this dose-response relationship was lost following adjustment for KOA risk factors of increased age, female sex, obesity, and previous knee injury, but also individuals’ leisure physical activity levels (Lo et al., 2019). The presence of such factors may negate the beneficial effects of swimming and possibly exercise in general, particularly increased age, female sex and obesity. Therefore, it may be that such individuals will require additional forms of KOA management such as topical NSAIDs.

However, the same cannot be said for leisure time physical activity levels, as it is not clear from studies whether high or low levels of leisure time physical activity negated the benefit of swimming. Moreover, it is possible that individuals who swam for longer may have been generally more physically active alongside swimming, driving the trend rather than swimming alone. Therefore, the optimal swimming dose cannot be assessed due to lack of investigation within the literature, although a self-selected dose, and three weekly 45-min sessions of moderate intensity swimming are beneficial.

### 3.6 Aquatic exercise

Aquatic exercise is a type of exercise whereby the participant performs exercise in water, such as aquatic walking, running, cycling, dancing, or resistance exercise (Yázigi et al., 2013a). The popularity of aquatic exercise is increasing, bringing the benefits of exercise on improving fitness and overall health (Kolasinski et al., 2019; Faíl et al., 2021), while water buoyancy allows for low impact joint motion (Kolasinski et al., 2019). As such, aquatic exercise has previously been recommended in the American College of Rheumatology KOA guidelines (Hochberg et al., 2012). Therefore, aquatic exercise in the management of KOA will be discussed to assess its therapeutic benefit and optimal dose.

#### 3.6.1 Aquatic exercise and symptom management

##### 3.6.1.1 Aquatic exercise and pain

Aquatic exercise reduced average weekly VAS pain by 69% over 6 weeks (Bressel et al., 2014), and prior to performing aquatic exercise, the same cohort completed a 4-week non-exercise control period, over which time average weekly VAS knee pain improved by 12% (Bressel et al., 2014). This double pre-test study design indicates with high internal validity that aquatic exercise improves knee pain compared to no exercise (Bressel et al., 2014). Knee pain following 8-weeks of aquatic exercise improved by 31%–43% on the WOMAC scale (Casilda-López et al., 2017; Taglietti et al., 2018), 23% on the KOOS pain scale (Lund et al., 2008), and 29% on the VAS scale (Taglietti et al., 2018), with 41% and 15% less pain on the VAS scale at rest and during walking respectively (Lund et al., 2008).

Over a 12-week period, knee pain remains improved with an 18% reduced KOOS pain score (Wang et al., 2011), although in a further 16-week study, pain only marginally reduced with a 5% reduction of KOOS pain score (Waller et al., 2017); although only those with a mild KL KOA severity were included, who had low baseline pain levels and thus small scope for improvement, thus potentially leading to only a marginal pain reduction.

##### 3.6.1.2 Aquatic exercise and physical function

Physical function improved by 17% on the WOMAC scale (Casilda-López et al., 2017), and 38% on the KOOS ADL scale following 8-week of aquatic exercise (Lund et al., 2008). Over 12-to-16-weeks KOOS ADL improved by 3%–4%, indicating small improvement in physical function (Wang et al., 2011; Waller et al., 2017). However, at baseline participants in both studies had high baseline KOOS ADL values, indicative of high baseline physical function, so likely had a little scope for improvement. This likely results from Wang et al. (2011), recruiting participants from the local community and sports centres and Waller et al. (2017), including only those with mild KOA, who therefore may not be reflective of clinical KOA populations. Moreover, physical function improves via walking speed increases by 5% (from 174 cm/s to 183 cm/s) (Waller et al., 2017), and 6MWT distance by 11%–20% (Casilda-López et al., 2017; Wang et al., 2011).

Aquatic exercise may also improve knee stiffness with WOMAC stiffness reducing by 9% after 8-weeks (Casilda-López et al., 2017), although no other studies have reported stiffness outcomes preventing definitive conclusions. However, total WOMAC score improved by 20%–62% over 8-weeks (Silvis et al., 2016; Casilda-López et al., 2017; Taglietti et al., 2018; Lim et al., 2010), indicative of the cumulative benefit of aquatic exercise on knee pain, physical function, and stiffness. Aquatic exercise also improved knee extensor and flexor strength by 6% and 12% respectively (Munukka et al., 2016). Furthermore, body mass index (BMI) was significantly reduced by 3% following 8 weeks of aquatic exercise (Lim et al., 2010), indicating a reduction of obesity which may increase over longer periods of aquatic exercise.

##### 3.6.1.3 Aquatic exercise and quality of life

The mental and physical SF-36 components have been improved by 16% and 13%–15% respectively (Taglietti et al., 2018; Lim et al., 2010), with 5%–10% improvement in KOOS QoL (Wang et al., 2011; Waller et al., 2017). Although, one study reported a 35% reduction on KOOS QoL scale, similar to that of the non-exercise control group (Lund et al., 2008). This may result from a short 8-week study period or differences in the type of aquatic exercise performed. However, over the same time period and when similarly performing aquatic resistance exercise, QoL was seen to improve in other studies (Taglietti et al., 2018; Lim et al., 2010). Variation in results may instead arise from differences in baseline participant characteristics such as KOA severity or QoL, however, may require further study.

Together, aquatic exercise may improve knee pain, stiffness, and physical function in those with KOA, including those with severe KOA (Waller et al., 2017; Taglietti et al., 2018), and those with KOA risk factors, including obesity (Waller et al., 2017; Casilda-López et al., 2017; Taglietti et al., 2018), female sex (Casilda-López et al., 2017; Waller et al., 2017), and older age (Taglietti et al., 2018; Lund et al., 2008; Wang et al., 2011). Although, the benefits of aquatic exercise over longer periods and on QoL are yet to be confirmed, warranting further study.

#### 3.6.2 Aquatic exercise: dose-response relationship

Three weekly 30-minute-high intensity aquatic exercise sessions improved average weekly VAS pain score by 69% over 6-weeks, with 100% session adherence (Bressel et al., 2014). A longer session duration, of three weekly 40-min sessions at a moderate intensity for 8-weeks improved total WOMAC score by 62% (Silvis et al., 2016). Therefore, due to greater VAS knee pain improvement than the cumulative improvement in WOMAC pain, stiffness, and physical function, a lower dose of three weekly 30-minute-high intensity sessions of aquatic may be more effective. Although, due to difference in assessment methods, such a definitive conclusion cannot be made.

However, three weekly 60-min moderate intensity sessions for 12-weeks improved KOOS pain and ADL by 18% and 4% respectively (Wang et al., 2011). While the same duration and frequency but at a high intensity for 16-weeks, improved KOOS pain and ADL by 5% and 4% respectively, with an adherence of 88% (Waller et al., 2017). Therefore, at this dose, a moderate exercise intensity may provide better symptom control, improving knee pain by a greater degree than a high exercise intensity, despite similar improvement to physical function. Therefore, conflicting results are seen regarding the dose-response of aquatic exercise, with variation in the symptom assessment methods across the doses limiting analysis and preventing identification of an optimal dose. However, current assessment shows the optimal weekly dose of aquatic exercise to be either three 30–40-minute-high intensity sessions or three 40–60-min moderate intensity sessions.

### 3.7 Resistance exercise

Resistance exercise is an exercise modality that aims to promote muscle strength through exercises that target different muscle actions. Resistance is applied to such action either using bodyweight, free weights, resistance bands or machines (Kraemer and Ratamess, 2004). Resistance exercise is a popular modality, performed by 44% of adults in England two or more times per week (Sport England, 2023), and linked to a 23% and 31% lower risk of all-cause and cancer mortality respectively (Stamatakis et al., 2017).

In relation to KOA, quadriceps muscle weakness is a known modifiable risk factor (Slemenda et al., 1997) but has also been shown to link to 15%–20% of lower limb function and 5% of OA related knee pain (Steultjens et al., 2001). Therefore, resistance exercise will subsequently be discussed, determining its therapeutic benefit and optimal dose in managing symptomatic KOA.

#### 3.7.1 Resistance exercise and symptom management

##### 3.7.1.1 Resistance exercise and pain

Lower limb resistance exercise over 8-week improved WOMAC pain score by 38%–55% (Jan et al., 2008; Lin et al., 2009; Chang et al., 2012); KOOS pain score by 21%–46% (Lund et al., 2008; McQuade and de Oliveira, 2011); and VAS pain by 18% when walking and 38% at rest (Lund et al., 2008). Knee pain remains reduced over 12-to-26-weeks with improved WOMAC pain score by 18%–54% (Jorge et al., 2014; Wang P. et al., 2016; Foroughi et al., 2011a; Foroughi et al., 2011b; DeVita et al., 2018; Baker et al., 2001); KOOS pain score by 14%–27% (Husted et al., 2021); and walking VAS pain by 57% (Wang P. et al., 2016). In addition, a combination of upper and lower body resistance exercise over a prolonged 78-week period reduced knee pain severity by 30%–39% on the WOMAC scale (Messier et al., 2021).

##### 3.7.1.2 Resistance exercise and physical function

When considering stiffness, lower limb resistance exercise improved WOMAC stiffness score by 37%–58% over 8-to-12-weeks (Chang et al., 2012; Jorge et al., 2014), and by 14%–52% over 24-to-26-weeks (Wang P. et al., 2016; Foroughi et al., 2011a; Foroughi et al., 2011b). While, WOMAC physical function improving by 43%–63% over 8-weeks (Jan et al., 2008; Lin et al., 2009; Chang et al., 2012), 38%–62% over 12-to-16-weeks (Jorge et al., 2014; DeVita et al., 2018; Baker et al., 2001), and 22%–30% over 26 weeks (Wang P. et al., 2016; Foroughi et al., 2011a). Furthermore, KOOS ADL improved by 19%–57% over 8-weeks (Lund et al., 2008; McQuade and de Oliveira, 2011), and 12%–20% over 12 weeks (Husted et al., 2021). Both upper and lower body resistance exercise over 78-weeks resulted in a 30%–44% improvement in WOMAC physical function (Messier et al., 2021).

In addition, functional measures of physical function were improved, with an 8%–23% increase in 6MWT distance (Wang P. et al., 2016; Messier et al., 2021), and a 16% increase in walking speed following resistance exercise (Lin et al., 2009). Muscle strength of those with KOA also improved, with knee extensor and flexor strength increasing by 9%–71% (Wang P. et al., 2016; Foroughi et al., 2011a; Baker et al., 2001; Husted et al., 2021; Messier et al., 2021), and 5%–57% (Jan et al., 2008; Wang P. et al., 2016; Foroughi et al., 2011a; Baker et al., 2001), respectively, with a 21% increase in STST repetitions (Chang et al., 2012).

Therefore, resistance exercise improved knee pain, stiffness, and physical function in those with KOA, including the elderly (Baker et al., 2001; Husted et al., 2021), obese (McQuade and de Oliveira, 2011; Jorge et al., 2014), and those with a severe KL KOA grade (DeVita et al., 2018; Husted et al., 2021). Furthermore, a combination of both upper and lower body resistance exercise were of similar benefit (Messier et al., 2021), although variation is seen in the degree of improvements for knee pain, stiffness, and physical function which may arise from differences in exercise dose, exercise selection, or training period.

##### 3.7.1.3 Resistance exercise and quality of life

Resistance exercise has shown improvements by 30% in CES-D score (Penninx et al., 2002), 19%–25% in KOOS QoL score (Husted et al., 2021), and 13% and 28% respectively within the SF-36 mental and physical domains (Jorge et al., 2014), indicative of improved QoL. However, KOOS QoL score reduced by 22%–25% in one study (Lund et al., 2008), indicating worse QoL, although may have resulted from the shorter 8-week treatment period.

##### 3.7.1.4 Resistance exercise and adverse events

Adverse events of greater knee pain were reported by 8%–32% of those with KOA (Lund et al., 2008; Jan et al., 2008; Messier et al., 2021), with 12% experiencing knee swelling following resistance exercise (Lund et al., 2008). Furthermore, when compared to the non-exercise control group, those performing resistance exercise reported a 5% greater incidence of falls (Messier et al., 2021), possibly due to those with KOA losing 10%–27% of their balance following a period of resistance exercise, increasing their fall risk (Lund et al., 2008).

#### 3.7.2 Resistance exercise: dose-response relationship

The maximum load an individual can lift for one repetition of an exercise is known as a “1-Repetition Maximum” (1RM) (Schoenfeld et al., 2021). The exercise intensity of resistance exercise is dependent on the number of repetitions performed at a relative load of the individuals’ 1RM (Schoenfeld et al., 2021), with “low” exercise intensity being 15 or more repetitions at less than 60% 1RM; “moderate” exercise intensity being 7–14 repetitions at 60%–79% 1RM; and “high” exercise intensity being 1 – 6 repetitions at 80% or greater of the 1RM (Schoenfeld et al., 2021; Day et al., 2004).

Low intensity lower limb resistance exercise performed twice weekly for 20-min over 8-weeks improved KOOS pain and ADL by 46% and 57% respectively with an adherence of 85% (Lund et al., 2008), while three weekly low intensity resistance exercise sessions for 10 exercise sets improved WOMAC pain by 38% and physical function by 43% over 8-weeks, with no adverse events (Jan et al., 2008). A greater dose of 12 low intensity lower limb exercise sets performed three times weekly for 26-weeks improved WOMAC knee pain, physical function, and stiffness by 18%–21%, 18%–22%, and 14%–18% respectively (Foroughi et al., 2011a; Foroughi et al., 2011b), with an adherence of 92% (Foroughi et al., 2011b). Three weekly sessions of 30 low intensity lower and upper body exercise sets for 78-weeks improved WOMAC pain by 39% and physical function by 44%, although adherence dropped to 69% (Messier et al., 2021). Therefore, regardless of low-intensity dose, similar degrees of improvement to knee pain and physical function are experienced. Although, at higher doses, adherence declines, however, the different doses were investigated over various time periods, likely influencing the dose-response trend.

Moderate intensity lower limb resistance exercise performed twice weekly for three exercise sets per session over 8-weeks improved WOMAC pain, stiffness, and physical function by 44%–55%, 58%, and 44%–54% respectively (Jan et al., 2008; Chang et al., 2012). Adherence was 96% (Chang et al., 2012), although 9% withdrew due to knee pain exacerbation (Jan et al., 2008). Three sets of moderate intensity lower limb resistance exercise was performed either two, four, or six sessions per week over 12-weeks in those with KOA (Husted et al., 2021). Two sessions per week most effectively improved knee pain and physical function, improving KOOS pain by 27% and ADL by 20%, with an adherence of 85% (Husted et al., 2021). Therefore, smaller doses of moderate intensity lower limb resistance exercise may be more effective, although were no more beneficial at any dose than low intensity lower limb resistance exercise.

High intensity lower limb resistance exercise performed for three weekly sessions of 18 exercise sets over 26 weeks improved WOMAC pain by 32%–33%, physical function by 31%–36%, and stiffness by 24%–26% (Foroughi et al., 2011a; Foroughi et al., 2011b). While over 78-weeks, three weekly sessions of 30 sets of high intensity lower and upper resistance exercise of which 18 sets were lower limb exercise, improved both WOMAC pain and physical function by 30% (Messier et al., 2021). Comparison within the same studies showed that high intensity was more effective at improving KOA symptoms than low intensity resistance exercise, although the difference was not significant, with an 11%–15%, 9%–18%, and 2%–8% greater improvement in WOMAC pain, physical function, and stiffness respectively (Foroughi et al., 2011a; Foroughi et al., 2011b). Furthermore, high intensity had a lower adherence at 85%, compared to 92% at a low intensity (Foroughi et al., 2011b).

However, within one study, low intensity resistance exercise improved knee pain and physical function by 9% and 14% more than high intensity resistance exercise, although not significantly. In addition, high intensity had a 3% lower adherence at 66%, and more adverse events, with 9% suffering body pain and falls and 6% suffering muscle strains. Whereas fewer adverse events were reported following low-intensity resistance exercise with 6%, 5%, and 2% reporting body pain, falls, and muscle strains respectively (Messier et al., 2021).

Therefore, KOA symptoms improve by a similar degree regardless of the intensity of resistance exercise. However, with a lower risk of adverse events and higher adherence, low-to-moderate intensity resistance exercise is most effective at managing knee pain, stiffness, and physical function of those with KOA. In addition, at this low-to-moderate intensity there was no further benefit at greater doses. Therefore, the optimal resistance exercise dose for KOA symptom management is either low intensity for 20 min duration or 10 sets of exercise performed two or three times weekly, or moderate intensity for three sets of exercise performed twice weekly.

## 4 Discussion

### 4.1 The optimal type of therapeutic exercise

Upon review of the literature, exercise of any modality is similarly beneficial in the management of KOA symptoms. Two systematic reviews, one of 72 studies (Vignon et al., 2006), and another of 10 studies (Smith et al., 2013), both investigated the effectiveness of different exercise types at managing KOA symptoms. In support of the present study, both showed similar benefit to knee pain, stiffness, and physical function in those with KOA regardless of the exercise modality (Vignon et al., 2006; Smith et al., 2013). Furthermore, Smith et al. (2013) concluded that the therapeutic stimulus of exercise may solely stem from joint movement, loading, and muscle contraction rather than the exercise type.

However, the present review highlights a limitation to the long-term effectiveness of exercise at managing KOA. It shows that despite similar adherence across the different exercise types, adherence fell to 50%–66% over up to 18-months (Ettinger et al., 1997; Messier et al., 2021), possibly reducing its long-term effectiveness. Therefore, to optimise future long-term effectiveness, further measures to promote exercise adherence may be required. A systematic review of 48 randomised controlled trials investigating different exercise modalities and doses, similarly showed exercise to improve KOA symptom management regardless of KOA severity, age, sex, or obesity (Juhl et al., 2014). The present review furthers this, describing how swimming provides KOA symptom control, although is yet to be investigated in those with severe KOA. In addition, limited research into running in those with KOA is seen within the literature. Therefore, running has only been seen to possibly improve knee pain frequency (Lo et al., 2018) and physical function (Lin et al., 2022), warranting further study to fully assess its therapeutic potential. However, runners with KOA should be encouraged to continue to run, but undertake injury prevention methods (Bosomworth, 2009), due to the risk of symptom exacerbation and KOA progression with knee joint injury (Driban et al., 2017).

Injury prevention methods include neuromuscular and proprioceptive training, stretching, plyometrics, core strengthening, and resistance training (Renstrom et al., 2008). Neuromuscular training involves functional weight-bearing movements involving multiple joints and muscle groups. This promotes sensorimotor joint control and functional joint stability, which reduces overall injury risk (Ageberg and Roos, 2015). Neuromuscular training alone has been shown to reduce rates of anterior cruciate ligament injuries of the knee by 50%, indicating its potential to reduce knee injury rates (Gagnier et al., 2012). Therefore, such injury prevention measures should be prescribed to those with KOA alongside their chosen type of exercise to prevent injury and thus allow the individual to exercise, improving their KOA symptom control.

Each exercise type had drawbacks reducing their relative effectiveness in different aspects of KOA management. Aquatic exercise minimally improved knee stiffness (Casilda-López et al., 2017), with conflicting influence on QoL for both aquatic (Yázigi et al., 2013a; Bressel et al., 2014; Casilda-López et al., 2017; Taglietti et al., 2018; Lund et al., 2008) and resistance exercise (Penninx et al., 2002; Lund et al., 2008; Jorge et al., 2014; Husted et al., 2021). Moreover, Tai Chi failed to reduce obesity, a key KOA risk factor (Wang et al., 2009). While cycling seemed improve knee pain, stiffness, and physical function but by a smaller magnitude than other exercise modalities, despite similar benefit to QoL, muscle strength (Liu et al., 2019; Mangione et al., 1999; Salacinski et al., 2012; Silvis et al., 2016; Keogh et al., 2018). Additionally, cycling failed to reduce obesity over a 12-week period, although did show the potential to do so over longer time periods (Alkatan et al., 2016), which then raises issues around adherence to regimens as already mentioned. Resistance exercise increased the fall risk by 5% (Messier et al., 2021), and a 10%–27% loss of balance (Lund et al., 2008), and therefore could be perceived as possibly leading to greater harm, particularly in the elderly with KOA who already are at a greater fall risk (Doré et al., 2015). Furthermore, fall risk in those with KOA may increase with therapeutic walking, although is likely no greater than the background fall risk (Kovar et al., 1992; Ettinger et al., 1997).

Despite the potential drawbacks of each exercise modality, greater benefit than risk to KOA management was seen, with marginal difference in outcomes between modes. The findings of this review support the NICE OA guidelines that “for all people with osteoarthritis, offer therapeutic exercise tailored to their needs (for example, local muscle strengthening, general aerobic fitness)” (National Institute for Health and Care Excellence, 2022), with all types of exercise being of similar benefit to all individuals with KOA.

Fundamentally, all individuals with KOA should continue the current exercise they do regardless of modality. While those who perform no exercise, should use this review to help inform shared decision making, in collaboration with clinicians, to tailor the exercise type to them.

### 4.2 The optimal dose of therapeutic exercise

Assessment of the dose-response relationship of each exercise modality to identify the optimal exercise dose found that at any dose knee pain, stiffness, and physical function were improved. Although, within the literature some specific doses of exercise were more effective than other exercise doses, thus being identified as “optimal”.

Moderate intensity walking three times weekly for 35-min was optimal (Rejeski et al., 1997), while for severe KOA, a weekly total of 50-min of moderate intensity walking over any number of sessions was optimal (Wallis et al., 2015). Three or four weekly sessions of 20–40-min of moderate intensity cycling was optimal for any individual with KOA (Keogh et al., 2018; Alkatan et al., 2016). For aquatic exercise, either three weekly, 30–40-minute-high intensity, or 40–60-min moderate-intensity aquatic exercise sessions was optimal (Silvis et al., 2016; Bressel et al., 2014; Wang et al., 2011). While for resistance exercise either low-intensity sessions for 20-min or 10 exercise sets performed two to three times weekly, or moderate-intensity sessions performed twice weekly for three exercise sets was optimal (Lund et al., 2008; Jan et al., 2008; Chang et al., 2012; Husted et al., 2021). The optimal Tai Chi dose could not be identified, although higher doses were more effective (Lee et al., 2018). Furthermore, due to a lack of investigation the optimal dose of running and swimming could not be assessed.

Therefore, the present study has shown that moderate intensity exercise performed three times weekly for 20–60-min is optimal for most exercise types. Prior work from Juhl et al. (2014) supports this finding, showing greater improvements in pain and physical function following three weekly exercise sessions. However, the present study finds exceptions to this such as in, walking where in those with severe KOA has a reduced optimal dose of a total weekly duration of 50-min at a moderate intensity over any number of sessions; cycling where four weekly sessions is also optimal; aquatic exercise where high intensity is also optimal; and resistance exercise where both a low intensity dose of 20-min or 10 exercise sets performed two to three times weekly or a moderate intensity dose performed twice weekly for three exercise sets is also optimal.

Additionally, the systematic reviews by Juhl et al. (2014) and Regnaux et al. (2015) found no greater benefit of high intensity exercise compared to lower intensities, further supported by the collated works within the present review. Although, in the present review, an exception to this was found, where when performing aquatic exercise, a high intensity was of equal benefit to lower intensities (Silvis et al., 2016; Bressel et al., 2014), likely due to the very low impact nature of aquatic exercise due to water buoyancy (Yázigi et al., 2013b).

However, methodological differences within the literature, including variation in participant characteristics, such as KOA severity, and the use of different assessment methods for symptoms, patient-centred outcomes, and modifiable risk factors, prevents reliable inter-study comparison. Therefore, the certainty by which conclusions regarding the optimal exercise modality and dose is limited. To draw definitive conclusions regarding the optimal exercise modality and dose for the management of KOA, future multi-arm randomised controlled trials including different exercise modalities and doses are required.

## 5 Strengths and limitations of review

A key strength to the present review is the assessment and comparison of the control of knee pain, stiffness, and physical function–all pertinent issues faced by people with KOA–following a range of different exercise modalities; making this a very patient oriented summary of the literature.

Additionally, the inclusion of key patient centred outcomes such as QoL, adherence, and modifiable KOA risk factors allow for greater assessment of the effectiveness of the different modalities at managing KOA. Therefore, this review adds to the literature by comparing exercise modalities to find the best at holistically managing KOA, rather than solely focusing on singular KOA symptoms. A further strength is the assessment of the dose-response relationship of each modality, giving greater understanding of how different exercise modalities can most effectively manage KOA symptoms. Ultimately, this study has given greater insight into whether an optimal modality and dose of exercise exists for the management of KOA, allowing those with KOA to better understand how to manage their condition with exercise, as recommended by the NICE OA management guidelines (National Institute for Health and Care Excellence, 2022).

Being a narrative literature review, the present study lacks a systematic methodology so is at risk of subjectivity and bias, such as selection or confirmation bias. Furthermore, with exercise interventions, participants cannot be blinded to the intervention arm to which they are assigned, due to the requirement for active participant involvement, bringing the possibility for performance bias where the perception of participants’ exercise allocation influences the results (Probst et al., 2016). Moreover, use of patient reported outcomes for assessment of knee pain, stiffness, and physical function can yield unreliable results due to self-report and recall bias, where responses can be attenuated or exaggerated (Althubaiti, 2016; Jeong et al., 2018). However, with no object biological marker for knee pain, patient reported assessment remains the most accurate and reliable method (Salaffi et al., 2004). Furthermore, the significance of monitoring KOA radiographically is disputed, with radiographic signs poorly reflecting an individual’s symptom severity (Bedson and Croft, 2008) and therefore, radiographic signs would make a poor indicator of KOA management. As a result, with no other reliable measures of KOA outcomes, patient reported symptom control remains the best indicator of an effective method of KOA management, as done in the present review.

When considering the subjective scales used, knee stiffness was assessed using the WOMAC stiffness score, which on testing has shown low validity and test-retest reliability (McConnell et al., 2001), so likely provides inaccurate knee stiffness assessment. However, the WOMAC score provides reliable, valid, and responsive assessment of pain and physical function (McConnell et al., 2001; White and Master, 2016). Such difference likely arises the WOMAC pain and physical function subscales being made up of five and seventeen questions respectively; while the stiffness subscale is composed of only two questions, resulting in a lower reliability following small variance in score and poor responsiveness to detect change in stiffness (McConnell et al., 2001). Furthermore, as already mentioned inter-study comparison of exercise types and doses was limited by variation between cohorts and study design, such as outcome assessment methods, leading to heterogeneity in the results, therefore hindering the ability to draw definitive conclusions.

Therefore, looking forward, future multi-arm, randomized controlled trials with different exercise modalities and doses are required to avoid unreliable inter-study comparisons. This will allow for definitive conclusions regarding the optimal modality and dose of therapeutic exercise, providing more reliable specific future exercise recommendations for those with KOA. Furthermore, with minimal research on running and swimming, further research to assess a range of doses of swimming and running in those with KOA is required to enable definitive conclusions regarding their benefit, optimal dose, and comparison to other modalities, which can guide future exercise prescription. A lower optimal walking dose was observed in those with severe KOA, highlighting the need for further research into the dose-response for other modalities in those with severe KOA. This would add to the literature by indicating whether, similar to walking, a lower optimal dose exists for other modalities, allowing for more specific exercise prescriptions for those with severe KOA.

A key finding of the present review was a drop in exercise adherence over longer treatment durations. Further investigation into long-term exercise interventions in those with KOA and the reasons for why sessions were missed would give insight into why adherence falls over time and help inform methods to mitigate this, improving long-term effectiveness. Additionally, the present review highlighted the potential of additional benefits following exercise performed in a group or supervised setting rather than unsupervised or individual exercise. Therefore, future study investigating whether group or supervised exercise is more effective is needed, which may further improve how exercise is used to manage KOA, such as through community-based exercise groups. Finally, the health economics of different exercise interventions remains to be investigated. This would provide insight into the most cost-effective exercise interventions for managing KOA, which may help inform decisions regarding community-based exercise interventions.

## 6 Conclusion

Therefore, together, clinicians and those with KOA should be confident that symptoms will be safely and effectively managed regardless of the exercise modality or dose. Therapeutic exercise of any type, and any dose, effectively manages knee pain, stiffness, and physical function in all individuals with KOA, supporting the NICE OA guidelines. This review highlights how different exercise types and doses influence KOA symptom management, while also assessing QoL, adherence, and the impact on modifiable KOA risk factors.

Exercise of any type proved beneficial to KOA management with similar benefit regardless of the type of exercise performed, thus with no optimal exercise modality identified. Assessment of the dose-response relationship to identify the optimal dose of the different exercise modalities, showed that any dose of regular exercise is beneficial. Although, for most modalities, moderate intensity exercise performed three times weekly for 20–60-min is most effective in managing KOA symptoms. Therefore, together, clinicians and those with KOA should be confident that symptoms will be safely and effectively managed regardless of the exercise modality or dose.

## Abbreviations

1RM, 1-Repetition Maximum; 6MWT, 6-Minute Walk Test; ADL, Activities of Daily Living; AIMS, Arthritis Impact Measurement Scale; BMI, Body Mass Index; CES-D, Centre for Epidemiologic Studies Depression; HCPs, Healthcare Professionals; HIIT, High Intensity Interval Training; ICOAP, Intermittent and Constant Osteoarthritis Pain; KL, Kellgren-Lawrence grade; KOA, Knee Osteoarthritis; KOOS, Knee injury and Osteoarthritis Outcome Score (KOOS); NICE, National Institute for Health and Care Excellence; NSAIDs, Non-Steroidal Anti-Inflammatory Drugs; OA, Osteoarthritis; QoL, Quality of Life; STST, Sit-To-Stand-Test; TKA, Total Knee Arthroplasty; VAS, Visual Analogue Scale; WOMAC, Western Ontario and McMaster Universities Osteoarthritis index.
